# Prevalence and Genetic Diversity of *Giardia duodenalis* and *Cryptosporidium* spp. among School Children in a Rural Area of the Amhara Region, North-West Ethiopia

**DOI:** 10.1371/journal.pone.0159992

**Published:** 2016-07-28

**Authors:** Aida de Lucio, Aranzazu Amor-Aramendía, Begoña Bailo, José M. Saugar, Melaku Anegagrie, Ana Arroyo, Beatriz López-Quintana, Derjew Zewdie, Zimmam Ayehubizu, Endalew Yizengaw, Bayeh Abera, Mulat Yimer, Wondemagen Mulu, Tadesse Hailu, Zaida Herrador, Isabel Fuentes, David Carmena

**Affiliations:** 1 Parasitology Service, National Centre for Microbiology, Majadahonda, Madrid, Spain; 2 National Centre of Tropical Medicine, Madrid, Spain; 3 Mundo Sano Foundation, Madrid, Spain; 4 Microbiology and Parasitology Service, La Paz-Carlos III Hospital, Madrid, Spain; 5 Department of Microbiology, Immunology and Parasitology, College of Medicine and Health Science, Bahir Dar, Ethiopia; Aga Khan University Hospital Nairobi, KENYA

## Abstract

**Backgroud:**

*Giardia duodenalis* and *Cryptosporidium* spp. are enteric protozoan causing gastrointestinal illness in humans and animals. Giardiasis and cryptosporidiosis are not formally considered as neglected tropical diseases, but belong to the group of poverty-related infectious diseases that impair the development and socio-economic potential of infected individuals in developing countries.

**Methods:**

We report here the prevalence and genetic diversity of *G*. *duodenalis* and *Cryptosporidium* spp. in children attending rural primary schools in the Bahir Dar district of the Amhara Region, Ethiopia. Stool samples were collected from 393 children and analysed by molecular methods. *G*. *duodenalis* was detected by real-time PCR, and the assemblages and sub-assemblages were determined by multilocus sequence-based genotyping of the glutamate dehydrogenase and β-giardin genes of the parasite. Detection and identification of *Cryptosporidium* species was carried out by sequencing of a partial fragment of the small-subunit ribosomal RNA gene.

**Principal Findings:**

The PCR-based prevalences of *G*. *duodenalis* and *Cryptosporidium* spp. were 55.0% (216/393) and 4.6% (18/393), respectively. A total of 78 *G*. *duodenalis* isolates were successfully characterized, revealing the presence of sub-assemblages AII (10.3%), BIII (28.2%), and BIV (32.0%). Discordant typing results AII/AIII and BIII/BIV were identified in 7.7% and 15.4% of the isolates, respectively. An additional five (6.4%) isolates were assigned to assemblage B. No mixed infections of assemblages A+B were found. Extensive genetic variation at the nucleotide level was observed within assemblage B (but no within assemblage A), resulting in the identification of a large number of sub-types. *Cryptosporidium* diversity was demonstrated by the occurrence of *C*. *hominis*, *C*. *parvum*, and *C*. *viatorum* in the population under study.

**Conclusions:**

Our data suggest an epidemiological scenario with an elevated transmission intensity of a wide range of *G*. *duodenalis* genetic variants. Importantly, the elevated degree of genetic diversity observed within assemblage B is consistent with the occurrence of intra-assemblage recombination in *G*. *duodenalis*.

## Introduction

After pneumonia, diarrhoeal diseases remain the second most common cause of death among children under five globally, with 49.6% of the associated deaths occurring only in Africa [1]. The protozoan *Giardia duodenalis* and *Cryptosporidium* spp. are the main non-viral causes of gastrointestinal illness with diarrhoea in humans, although infections by these pathogens are rarely fatal. It is estimated that approximately 200 million people per year experience symptomatic giardiasis whereas cryptosporidiosis accounts for up to 20% of all cases of childhood diarrhoea in low-income countries [2,3]. In combination with diarrhoea, *G*. *duodenalis* and *Cryptosporidium* infections may cause malabsorption and long-term nutritional deficit leading to stunting, growth and cognitive retardation, and failure to thrive during childhood and adolescence [4,5]. Due to their associated disease burden and considerable socio-economic consequences, both *Giardia* and *Cryptosporidium* joined the Neglected Disease Initiative launched by the World Health Organisation (WHO) in 2004 [6].

Children with decreased intake of food and poor overall health are more exposed to giardiasis and cryptosporidiosis. Therefore, a strong interrelation exists between the incidence and severity of these diseases and the socio-economic status and education level of the affected population [7]. Sanitation and hygiene practices also play a critical role in the transmission of *Giardia* and *Cryptosporidium*. Indeed, interventions aiming to improve accessibility to clean water and latrines have been demonstrated very effective in reducing the prevalence of intestinal helminths and protozoa in endemic areas including Ethiopia [8–10].

The taxonomy of *G*. *duodenalis* and *Cryptosporidium* is far from completed and still subjected to intense debate. *G*. *duodenalis* is currently regarded as a multi-species complex divided into eight distinct genetic assemblages (A to H) that show different host range and specificity, virulence, and geographical distribution. Of them, only assemblages A and B are considered to be pathogenic to humans, although they can be also found in other mammals including pets and livestock and pose, therefore, zoonotic potential [11,12]. Regarding *Cryptosporidium*, between 26 and 30 species have been proposed as taxonomically valid based on morphological, biological, and molecular features. Human infections are mostly caused by the highly host-adapted *C*. *hominis* or the more generalist (and zoonotic) *C*. *parvum*, although at least eight additional *Cryptosporidium* species have been less frequently or sporadically detected in humans [13–15].

As in other sub-Saharan African countries, diarrhoea is a leading cause of morbidity and mortality among young children in Ethiopia, where the total number of annual deaths due to diarrheagenic illness has been estimated at more than 73,000 children [7]. *G*. *duodenalis* and *Cryptosporidium* spp. have been frequently detected in Ethiopian paediatric populations. Population-based coprological studies conducted in different geographical regions of the country reported prevalences of giardiasis and cryptosporidiosis in the range of 12–35% and 7–12%, respectively, in school [10], hospital [16], and community [8,17,18] settings. In addition, *G*. *duodenalis* and *Cryptosporidium* spp. have also been identified at variable infection rates in individuals with diarrhoea seeking medical care [19–22], food handlers [23], and community [24], adult HIV patient [25,26], and prison inmate [27] populations.

In contrast to the comparative abundance of *G*. *duodenalis* and *Cryptosporidium* spp. prevalence data, very few molecular studies have been carried out to ascertain the current diversity and frequency of occurrence of the genotypes and sub-types of these enteric pathogens in Ethiopian human populations [22,28,29]. Available molecular data is, therefore, restricted to the relatively low number of isolates obtained and successfully typed in these studies. In an early typing survey involving *G*. *duodenalis*-positive samples from different community settings throughout the country, assemblages A and B were identified in 52% and 22% of the isolates, respectively. Additionally, mixed infections A+B and A+F were also detected in 14% and 12% of the isolates, respectively [28]. A preliminary molecular study conducted in a rural hospital in Southern Ethiopia revealed the presence of *G*. *duodenalis* assemblage B, but not assemblage A, in individuals seeking medical attention with symptomatic giardiasis [22]. Similarly, *C*. *parvum* was the *Cryptosporidium* species most frequently (97%) identified in patients with diarrhoea in a multi-regional study comprising nine different geographical areas of Ethiopia [29]. *C*. *hominis* has also been detected in a limited number of human isolates [22,29].

In an attempt to improve and expand our current knowledge on the epidemiological and molecular situation of human giardiasis and cryptosporidiosis in Ethiopia, we present here a thorough assessment of the prevalence and genetic diversity of *G*. *duodenalis* and *Cryptosporidium* spp. in a large population of primary school children in a rural area of the Amhara Region.

## Methods

### Ethical statement

The study design and consent procedures of this survey have been approved by the Amhara National Regional State Health Bureau Ethics Review Committee in Bahir Dar (Ethiopia). The export permit for of the obtained DNA samples was obtained from the Ethiopian Institute of Biodiversity in Addis Ababa. Gathered or generated socio-demographic or clinical data were conveniently anonymized prior to any analysis to preserve the identity of the participants.

### Area of study

This survey was conducted in Bahir Dar, a district (*woreda*) belonging to the West Gojjam Zone in the Amhara Region in north-western Ethiopia. With an area of 1,443·km^2^, the Bahir Dar district is divided in 32 municipalities (*kebeles*), has a total population 182,676 with a male/female ratio of 1.05, a population density of 133.6 inhabitants·km^-2^, and an average household size of 4.3 [30]. The region is part of the Ethiopian highlands, between 1,500 and 2,300 meters elevation, and is characterized by a temperate climate with a relatively short rainfall season extending from about June to October and averaging over 1,200 mm annually. The main economic activity of the population is agriculture. Predominant crops include wheat, teff, barley, maize, sorghum, and chickpeas.

### Study design

This study is part of a research program conducted by the Mundo Sano Foundation (Spain-Argentina) in partnership with the Carlos III Health Institute (Spain) that holds collaboration agreements with local institutions including the Regional Health Bureau and the Bahir Dar University (Ethiopia). The main goal of this program was the collection of baseline prevalence data on soil transmitted helminths and other intestinal parasites in the Bahir Dar district, and, if required, planning for control interventions. Paediatric populations were preferentially sampled in this study because children are more susceptible than the general population to parasitic infections, and therefore, are considered good targets for assessing the epidemiological status of a specific disease in a given area.

A cross-sectional survey was conducted among children attending eight rural public primary schools of the Bahir Dar district between October and November 2013. Permission were obtained from the Regional Education Bureau and the local education authorities. School representatives were then contacted and asked for collaboration. Informative meetings were held with teachers to explain the aim of the study and the procedures involved. Appointments were then scheduled at suitable times to provide the participating children with a pre-labelled sampling kit including a sterile polystyrene flask for the recovery of the stool sample and a written informed consent (in Amharic) to be signed by the parents or legal guardians. Information regarding the purpose of the project and instructions on how to take the sample correctly was also issued to each participant.

### Human stool sample collection

Distribution and collection of sampling kits was arranged by the collaborating teachers at the scheduled times. Signed informed consents and basic socio-demographic data including school, age, and gender were obtained from all participants and carefully checked for completeness and accuracy. Obtained stool samples were immediately transported to the College of Medicine and Health Science, Bahir Dar University (Ethiopia), and processed within the same day of collection.

### DNA extraction and purification

In order to improve parasite recovery and identification, an aliquot (~1 g) of fresh faecal material was processed using the stool concentration system BioParaprep Mini^®^ (Leti Diagnósticos, Barcelona, Spain) according to the manufacturer’s instructions. Total DNA was extracted from 200 mg of concentrated faecal material using the QIAamp^®^ DNA stool mini test kit (Qiagen, Hilden, Germany). Purified DNA samples (200 μL) were stored at –20°C and shipped to the National Centre for Microbiology, Majadahonda, Spain, for further molecular analysis.

### Molecular detection of *Giardia duodenalis*

A real-time PCR was initially used for the specific detection of *G*. *duodenalis* in faecal samples [31]. This assay targeted a 62-bp region of the small subunit ribosomal RNA *(SSU* rRNA) gene of the parasite using the primer pair Gd-80F and Gd-127R (S1 Table) and the probe (6-carboxyfluorescein[FAM]-5´-CCCGCGGCGGTCCCTGCTAG-3´-black hole quencher 1 [BHQ1]). This gene is particularly suited for screening large number of samples because of its multi-copy nature. Amplification reactions were performed in a volume of 25 μL containing 3 μL of genomic DNA, 500 nM of each primer, 200 nM of probe, and TaqMan^®^ Gene Expression Master Mix (Applied Biosystems, California, USA). We adopted the amplification protocol for TaqMan^®^ recommended by the manufacturer, consisting on an initial hold step of 2 min at 55°C and 15 min at 95°C followed by 45 cycles of 15 s at 95°C and 1 min at 60°C. Appropriate positive, negative, and inhibition controls were routinely included in each round of real-time PCR assays. Amplification and detection of parasitic DNA were performed on a Corbett Rotor-Gene 6000 real-time PCR cycler (Qiagen Corbett, Hilden, Germany). Rotor Gene 6000 Series software version 1.7 was used for data analysis. Fluorescence (510 nm) was measured at the end of the annealing step of each cycle. The ramping of the machine was 10°C/s in every step.

### Molecular characterization of *Giardia duodenalis* isolates

*G*. *duodenalis* isolates that tested positive by real-time PCR were subsequently analysed by multi-locus genotyping (MLG) using two gene loci: glutamate dehydrogenase (*GDH*) and ß-giardin (*BG*). The amplification of the *GDH* gene was performed by a semi-nested PCR with minor modifications [32]. The primer pair GDHeF and GDHiR (S1 Table) was used in the primary PCR with 5 μL of genomic DNA. Five μL of PCR product from the primary reaction was added to the secondary PCR containing the primer pair GDHiF and GDHiR (S1 Table) to yield a 432-bp fragment. Final concentrations of reagents in PCR reaction mixtures were as follow: 500 nM of each primer, 200 μM of each dNTP, 1.5 mM MgCl_2_, 2.5 units of *Taq* DNA polymerase, and Reaction Buffer in a final volume of 25 μL. The primary and secondary PCR reactions were carried out as follows: 1 cycle of 95°C for 3 min, followed by 35 cycles of 95°C for 30 s, 55°C for 30 s and 72°C for 1 min. A final extension of 72°C for 7 min and a 4°C hold was used.

The amplification of the *BG* gene was performed using a nested PCR with minor modifications [33]. The primer pair G7_F and G759_R (S1 Table) was used in the primary PCR with 3 μL of geomic DNA. Three μL of PCR product from the primary reaction were added to the secondary PCR containing the primer pair G99_F and G609_R (S1 Table) to yield a 511-bp fragment. Final concentrations of reagents in PCR reaction mixtures (25 μL) included 400 nM of each primer, 200 μM of each dNTP, 1.5 mM MgCl_2_, 2.5 units of *Taq* DNA polymerase, and Reaction Buffer. The primary PCR reaction was carried out with the following amplification condition: 1 cycle of 95°C for 7 min, followed by 35 cycles of 95°C for 30 s, 65°C for 30 s, and 72°C for 1 min. A final extension of 72°C for 7 min and a 4°C hold was used. Cycling parameters for the secondary PCR reaction were the same as above except that the annealing temperature was 55°C.

### Molecular detection and characterization of *Cryptosporidium* species

Identification of *Cryptosporidium* species was carried out by a nested-PCR assay targeting the *SSU* rRNA gene of the parasite [34]. In the first round of PCR, 3 μL of DNA sample were amplified using the primer pair 18SiCF2 and 18SiCR2 (S1 Table). Three μL of PCR product from the primary reaction was added to the secondary PCR containing the primer pair 18SiCF1 and 18SiCR1 to amplify a 587-bp fragment. PCR reaction mixtures consisted of 300 nM of each primer, 200 μM of each dNTP, 1.5 mM MgCl_2_, 2.5 units of *Taq* DNA polymerase, and Reaction Buffer in a final volume of 50 μL. Both PCR reactions were carried out as follows: 1 cycle of 94°C for 3 min, followed by 35 cycles of 94°C for 40 s, 50°C for 40 s and 72°C for 1 min. A final extension of 72°C for 10 min and a 4°C hold terminated both PCR reactions.

All conventional *G*. *duodenalis* and *Cryptosporidium* PCR reactions were carried out using BIOTAQ^™^ DNA polymerase (Bioline GmbH, Luckenwalde, Germany) on a 2720 thermal cycler (Applied Biosystems, California, USA). Appropriate positive and negative controls were routinely included in each round of PCR. PCR products were visualized on 2% D5 agarose gels (Conda, Madrid, Spain) stained with Pronasafe nucleic acid staining solution (Conda, Madrid, Spain). In order to confirm *G*. *duodenalis* assemblages and *Cryptosporidium* species, positive-PCR products were directly sequenced in both directions using the same internal primer sets as in the respective PCR assays.

### Data analyses

Raw sequencing data in both forward and reverse directions were viewed using the Chromas Lite version 2.1 sequence analysis program (http://chromaslite.software.informer.com/2.1/). Special attention was paid to the identification of heterozygous sites (double peaks) in the electropherograms. The BLAST tool (http://blast.ncbi.nlm.nih.gov/Blast.cgi) was used to compare nucleotide sequences with sequences deposited in the NCBI, GiardiaDB (http://giardiadb.org/giardiadb/), and CryptoDB (http://cryptodb.org/cryptodb/) databases. The resulting DNA consensus sequences were aligned to reference sequences using ClustalW in MEGA version 6.0 (http://www.megasoftware.net/) to determine *G*. *duodenalis* assemblages and *Cryptosporidium* species. Phylogenetic analyses, based on the Neighbour-Joining method, were performed using the same software [35]. Sequences including heterozygous (di-nucleotide) sites were excluded from the analyses in order to avoid distorting the topology of the phylogenetic trees.

The Chi-square test was used to compare parasite infection rates by school, gender, and age group. A probability (*P*) value < 0.05 was considered evidence of statistical significance. The paired Student's "t" test was used for analysing the significance (*P* value < 0.01) between real-time PCR cycle threshold (Ct) values and the detection of *G*. *duodenalis* infections categorized according to different age groups.

## Results

### Detection of *G*. *duodenalis*

**Fig 1:**
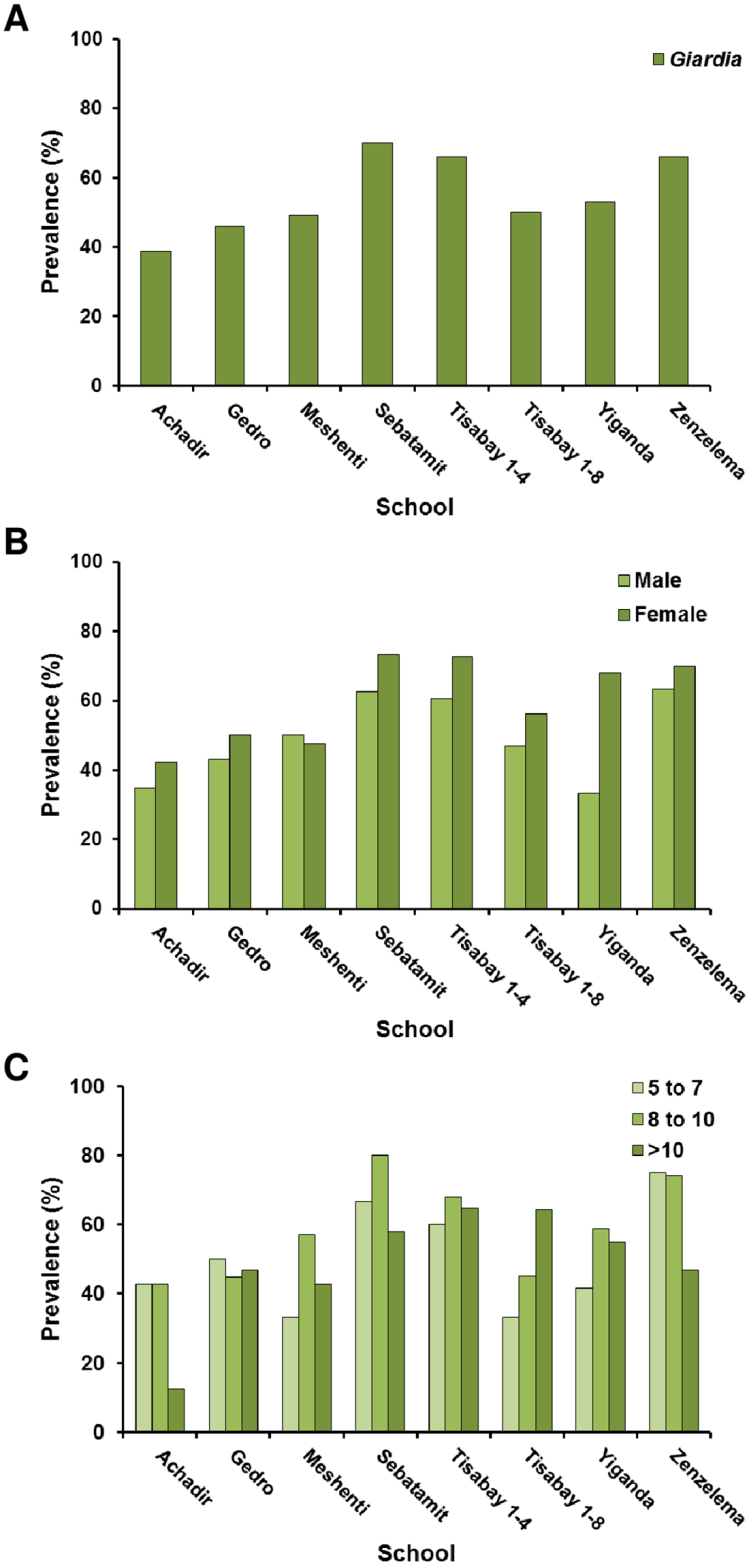
Prevalence of *Giardia duodenalis* in children attending rural schools in the Bahir Dar district, Amhara Region, Ethiopia. Results were segregated by school (A), gender (B), and group of age (C).

**Fig 2:**
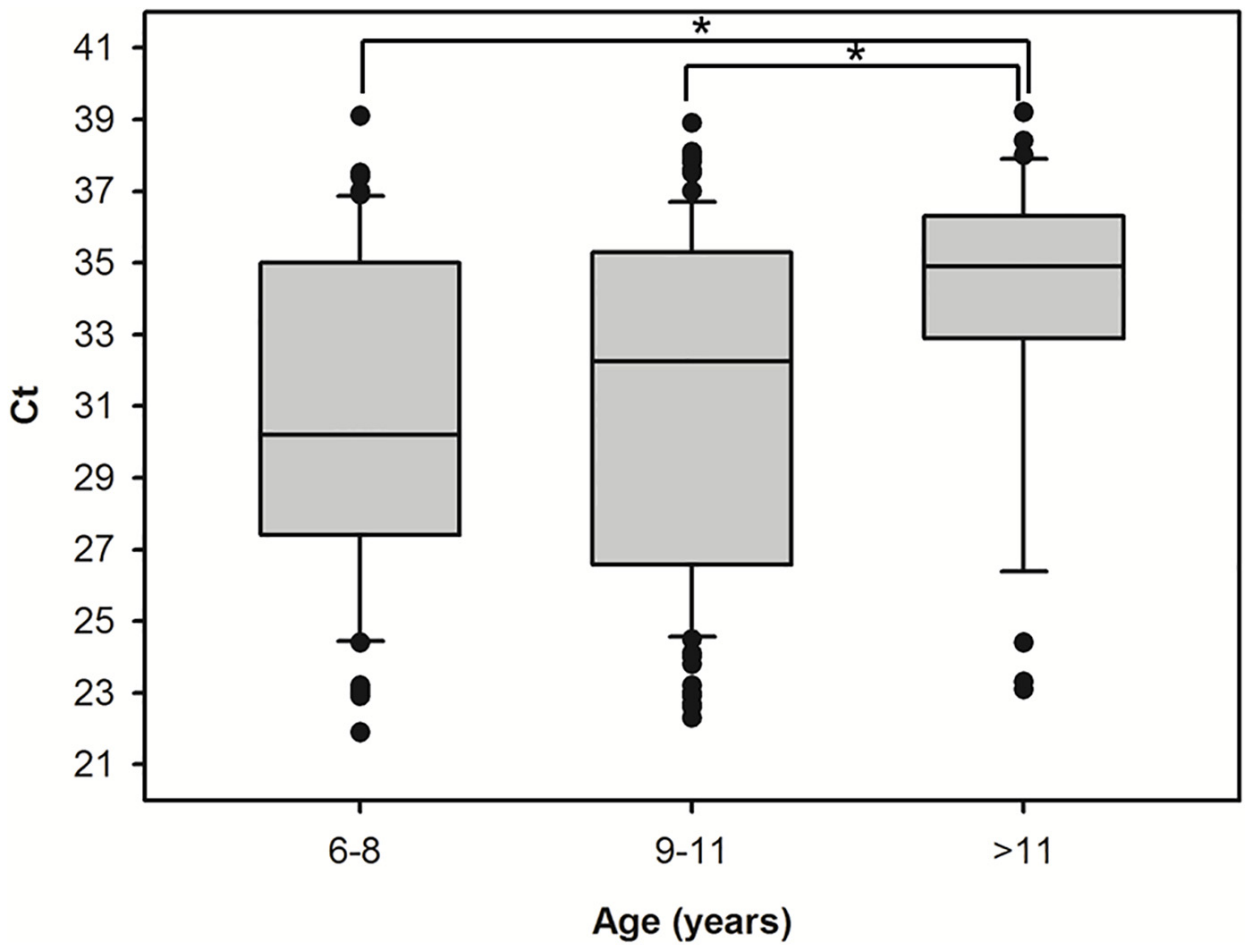
Median real-time PCR cycle threshold (Ct) values from *Giardia duodenali*s positive samples by age group in children aged 6–15 years in the Amhara Region, Ethiopia. Statistical significance (P < 0.01) is indicated by asterisks.

A total of 393 stool samples from children attending eight representative rural schools in the Bahir Dar district were collected during this cross-sectional study. Overall, stool samples were obtained from 8.6% of the total population of primary school children in this area (Table 1). The male/female ratio was 1.12. The age range was from 6 to 15 years (mean: 9.69; SD: 1.80). The real-time PCR-based *G*. *duodenalis* infection rate was estimated at 55.0% (95% CI: 50.1–59.9%), although this figure was highly variable (from 38.8–70.0%) among the primary schools visited (Fig 1, panel A). Typically, females were found more frequently infected by *G*. *duodenalis* than males (61.1% vs. 49.5%, respectively) (Fig 1, panel B), this difference being statistically significant (P < 0.05, χ^2^ = 5.29). On the contrary, *G*. *duodenalis* infections were homogeneously distributed among all the different age groups considered in this survey (Fig 1 panel C), and no statistically significant differences could be demonstrated among them (P > 0.05, χ^2^ = 2.53). Real-time PCR-positive samples had Ct values ranging from 21.9 to 39.2 (mean: 31.50; SD: 4.70). A significant correlation between Δ-Ct values and children group of age was demonstrated, with older children tending to provide higher Ct values than younger children (Fig 2).

**Table 1:** Prevalence and assemblage frequencies of *Giardia duodenalis* in children attending rural schools in the Bahir Dar district, Amhara Region, Ethiopia, 2013.

School	Children					Assemblage^b^	
	Total (n)	Sampled (n)	*Giardia*-positive (n)^a^	%	Isolates typed (n)	A (%)	B (%)
Achadir	273	49	19	38.8	9	0 (0.0)	9 (100)
Gedro	413	48	22	45.8	9	1 (11.1)	8 (88.9)
Meshenti	744	49	24	49.0	11	0 (0.0)	11 (100)
Sebatamit	448	50	35	70.0	8	3 (37.5)	5 (62.5)
Tisabay 1–4	555	50	33	66.0	8	1 (12.5)	7 (87.5)
Tisabay 1–8	1064	48	24	50.0	11	1 (9.1)	10 (90.9)
Yiganda	102	49	26	53.1	7	4 (57.1)	3 (42.9)
Zenzelema	940	50	33	66.0	15	4 (26.7)	11 (73.3)
Total	4,539	393	216	55.0	78	14 (17.9)	64 (82.1)

^a^Samples positive by real-time PCR.

^b^Number and frequency of *G*. *duodenalis* sub-assemblages characterized at the *GDH* and/or *BG* loci.

### PCR amplification and sequencing of *G*. *duodenalis* at the *GDH* and *BG* loci

Among 216 real-time PCR products that tested positive for *G*. *duodenalis*, 73 (33.8%) were successfully amplified at the *GDH* locus, and 53 (24.5%) at the *BG* locus, respectively. MLG data were available for 48 typing results, whereas 25 isolates were characterized at the *GDH* locus only and 5 isolates at the *BG* locus only. Overall, assemblages A and B were detected in 17.9 (14/78) and 82.1 (64/78), respectively, of the *G*. *duodenalis* isolates typed at these markers (Table 1).

Sequence analysis of the 73 *GDH*-PCR products allowed the identification of 14 sub-assemblages AII, 23 sub-assemblages BIII, and 24 sub-assemblages BIV. Discordant genotype results BIII/BIV were obtained in 12 additional *G*. *duodenalis* isolates (Table 2). Similarly, sequence analysis of the 53 *BG*-PCR products revealed the presence of two sub-assemblages AII (confirming the typing results obtained at the *GDH* locus), six sub-assemblages AIII (all of them previously assigned to sub-assemblage AII at the *GDH* locus), and 45 assemblages B, of which 40 were also confirmed at the *GDH* locus (Table 2).

**Table 2:** Sub-assemblage frequencies of *Giardia duodenalis* in children attending rural schools in the Bahir Dar district, Amhara Region, Ethiopia, 2013.

School	Isolate	Results at the *GDH* locus	Results at the *BG* locus	Assigned genotype
Achadir	AC-05	BIV	B	BIV
	AC-06	BIII	No amplification	BIII
	AC-09	No amplification	B	B
	AC-11	BIII/BIV	No amplification	BIII/BIV
	AC-13	BIII	B	BIII
	AC-15	BIII	B	BIII
	AC-35	BIII/BIV	B	BIII/BIV
	AC-42	BIII	No amplification	BIII
	AC-47	BIV	B	BIV
Gedro	GE-01	BIV	B	BIV
	GE-03	BIV	B	BIV
	GE-08	BIII/BIV	B	BIII/BIV
	GE-10	BIII/BIV	No amplification	BIII/BIV
	GE-21	BIII/BIV	B	BIII/BIV
	GE-30	BIV	B	BIV
	GE-34	BIII/BIV	No amplification	BIII/BIV
	GE-40	AII	No amplification	AII
	GE-47	BIII/BIV	No amplification	BIII/BIV
Meshenti	ME-06	BIII	No amplification	BIII
	ME-07	BIV	B	BIV
	ME-08	BIII	B	BIII
	ME-14	BIII	No amplification	BIII
	ME-19	BIII/BIV	B	BIII/BIV
	ME-20	BIII	B	BIII
	ME-27	BIV	No amplification	BIV
	ME-30	BIII	No amplification	BIII
	ME-32	BIV	B	BIV
	ME-38	BIII	No amplification	BIII
	ME-50	BIV	B	BIV
Sebatamit	SE-07	AII	No amplification	AII
	SE-10	AII	AIII	AII/AIII
	SE-11	BIV	No amplification	BIV
	SE-12	AII	AIII	AII/AIII
	SE-16	BIV	No amplification	BIV
	SE-31	BIII/BIV	B	BIII/BIV
	SE-40	BIV	B	BIV
	SE-46	BIV	B	BIV
Tisabay 1–4	TS-06	BIII	B	BIII
	TS-09	BIV	B	BIV
	TS-24	BIII	B	BIII
	TS-30	BIV	B	BIV
	TS-34	BIII	No amplification	BIII
	TS-36	BIV	B	BIV
	TS-45	BIV	B	BIV
	TS-50	AII	No amplification	AII
Tisabay 1–8	TB-04	BIII	B	BIII
	TB-15	BIV	No amplification	BIV
	TB-17	BIII	B	BIII
	TB-21	No amplification	B	B
	TB-25	BIII/BIV	B	BIII/BIV
	TB-30	AII	No amplification	AII
	TB-31	BIII/BIV	No amplification	BIII/BIV
	TB-33	BIV	B	BIV
	TB-37	BIV	No amplification	BIV
	TB-38	BIV	B	BIV
	TB-45	BIV	B	BIV
Yiganda	YI-04	AII	AIII	AII/AIII
	YI-11	BIII	B	BIII
	YI-13	BIII	B	BIII
	YI-32	AII	AIII	AII/AIII
	YI-36	AII	AIII	AII/AIII
	YI-38	AII	AIII	AII/AIII
	YI-47	BIII	B	BIII
Zenzelema	ZE-05	No amplification	B	B
	ZE-10	AII	No amplification	AII
	ZE-11	AII	No amplification	AII
	ZE-16	BIII	B	BIII
	ZE-17	BIV	B	BIV
	ZE-21	BIV	B	BIV
	ZE-25	AII	AII	AII
	ZE-26	BIII	No amplification	BIV
	ZE-32	No amplification	B	B
	ZE-33	BIII	B	BIII
	ZE-36	No amplification	B	B
	ZE-37	BIII/BIV	No amplification	BIII/BIV
	ZE-38	BIII	B	BIII
	ZE-40	BIII	B	BIII
	ZE-46	AII	AII	AII

Failure to amplify real-time PCR-positive samples at the *GDH* and/or *BG* genes was found to be strongly associated with high Ct values, as demonstrated by the fact that out of the 132 (61.1%) samples with Ct values >30 only five and two could be successfully typed by *GDH*-PCR and *BG*-PCR, respectively.

### Molecular characterization of *G*. *duodenalis* isolates at the *GDH* locus

Good quality sequencing data with clear electropherograms that had low background noise and distinct sharp peaks for each nucleotide along the sequence were used for the multiple sequence alignment analyses. Special attention was paid to the identification of di-nucleotide (double peak) sites in the sequencing trace.

Regarding the typing of *GDH* amplicons, alignment analysis of the 14 isolates assigned to *G*. *duodenalis* sub-assemblage AII allowed the identification of a 399-bp fragment that showed 100% identity with the partial *GDH* gene equivalent to positions 80–478 of the corresponding reference sequence (GenBank accession number L40510). This single AII genotype was submitted to GenBank under accession number KP899829.

Alignment analysis of the 22 isolates characterized as *G*. *duodenalis* sub-assemblage BIII with reference sequence AF069059 for the partial *GDH* gene allowed the identification of a 395-bp fragment, equivalent to positions 44–438 of AF069059. None of these isolates had sequences identical to the corresponding reference sequence, differing from it by one to eight single-nucleotide polymorphisms (SNPs). A total of 20 (seven novel and 13 known) sub-types were identified and submitted to GenBank under accession numbers KP899832 to KP899851 (Table 3). All SNPs identified corresponded to transition (C ↔ T or A ↔ G) mutations, with mixed bases (double peaks) accounting for 67.9% (53/78) of the total. Only three point mutations were associated to amino-acid changes at the protein level (Table 3).

**Table 3:** Diversity and frequency of single-nucleotide polymorphisms displayed by *Giardia duodenalis* sub-assemblage BIII isolates at the glutamate dehydrogenase locus (partial sequence between positions 44 to 438) identified in the present study. Novel sub-types were shown underlined. Point mutations inducing amino-acid substitutions were marked as superscript indicating the amino-acid change.

		Nucleotide at position of reference sequence AF069059																								
		64	78	87	99	132	147	150	174	180	181	204	219	237	243	309	318	324	330	336	351	372	375	396	402	424
		C	C	C	C	C	T	G	C	C	G	C	T	T	C	C	G	A	C	C	C	G	C	C	G	G
Sub-type	Number of isolates																									
KP899832	1	Y^1^	Y	Y	.	.	Y	.	.	.	.	.	.	.	.	T	.	.	.	.	.	.	.	.	.	.
KP899833	1	.	.	T	.	.	C	A	.	.	.	.	.	.	.	.	.	.	.	.	.	.	.	.	.	A^3^
KP899834	1	.	.	T	.	.	.	.	.	.	.	.	.	.	.	.	.	.	Y	.	Y	.	.	.	.	.
KP899835	1	.	.	.	Y	.	Y	.	.	.	.	.	Y	Y	.	.	.	.	T	.	.	.	.	.	R	.
KP899836	1	.	.	.	Y	.	Y	R	.	.	.	.	Y	Y	.	.	.	.	T	.	.	.	.	Y	R	.
KP899837	1	.	.	.	T	.	.	R	.	.	R^2^	.	.	.	.	.	.	.	.	.	.	.	.	.	.	.
KP899838	1	.	.	.	T	.	.	.	.	.	.	Y	.	.	Y	.	R	.	.	.	.	.	.	.	.	.
KP899839	1	.	.	.	Y	.	.	.	.	.	.	.	.	.	Y	Y	.	.	Y	.	Y	.	.	.	R	.
KP899840	1	.	.	.	.	T	C	A	T	.	.	T	.	.	.	.	.	G	.	.	.	.	.	.	.	.
KP899841	1	.	.	.	.	.	Y	R	.	.	.	.	.	.	.	.	.	.	.	.	.	.	.	.	R	.
KP899842	1	.	.	.	.	.	Y	.	.	Y	.	.	Y	Y	.	.	.	.	.	.	.	.	.	.	.	.
KP899843	1	.	.	.	.	.	Y	.	.	.	.	.	Y	Y	.	.	.	.	.	.	.	.	.	.	.	.
KP899844	1	.	.	.	.	.	Y	.	.	.	.	.	Y	.	.	Y	.	.	Y	Y	.	.	.	.	.	.
KP899845	1	.	.	.	.	.	Y	.	.	.	.	.	Y	.	.	.	.	.	Y	.	.	.	.	.	R	.
KP899846	1	.	.	.	.	.	C	.	.	.	.	.	.	.	.	T	.	.	T	.	.	.	.	.	.	.
KP899847	1	.	.	.	.	.	.	.	.	.	.	.	Y	Y	.	.	.	.	.	.	.	.	.	.	.	.
KP899848	1	.	.	.	.	.	.	.	.	.	.	.	.	Y	.	.	.	.	Y	.	.	.	.	.	R	.
KP899849	1	.	.	.	.	.	.	.	.	.	.	.	.	.	T	.	.	.	.	.	.	A	T	.	.	.
KP899850	3	.	.	.	.	.	.	.	.	.	.	.	.	.	.	T	.	.	.	.	.	.	.	.	.	.
KP899851	1	.	.	.	.	.	.	.	.	.	.	.	.	.	.	T	.	.	.	T	.	.	.	.	.	.

R: A/G; Y: C/T.

^1^ p.L22F.

^2^ p.D61N.

^3^ p.A142T.

Similarly, alignment of the 24 isolates typed as *G*. *duodenalis* sub-assemblage BIV with reference sequence L40508 for the partial *GDH* gene generated a 388-bp fragment, corresponding to positions 80–467 of L40508. As in the case of BIII samples, a very high degree of genetic polymorphism was seen among the obtained BIV isolates. This variability at the nucleotide level resulted in the identification of 23 (5 novel and 18 known) sub-types that were deposited in GenBank under accession numbers KP899852 to KP899874. None of them exhibited 100% similarity with the reference sequence L40508, from which they differed by 2 to ten SNPs (Table 4). Out of the 120 SNPs detected, 118 were transition mutations and the remaining two transversion (purine ↔ pyrimidine) mutations. Double peaks were identified in 60.0% (72/120) of the cases. All the SNPs found except two corresponded to silent mutations with no effect in the amino-acid sequence of the protein (Table 4).

**Table 4:** Diversity and frequency of single-nucleotide polymorphisms displayed by *Giardia duodenalis* sub-assemblage BIV isolates at the glutamate dehydrogenase locus (partial sequence between positions 80 to 467) identified in the present study. Novel sub-types were shown underlined. Transversion mutations were highlighted in bold. Point mutations inducing amino-acid substitutions were marked as superscript indicating the amino-acid change.

		Nucleotide at position of reference sequence L40508																											
		87	105	123	135	183	186	201	228	231	255	258	273	282	291	295	300	327	336	345	366	372	387	396	423	432	438	450	462
		C	C	C	T	T	G	G	C	G	C	C	C	G	C	G	C	C	T	C	T	C	T	C	C	C	A	A	T
Sub-type	Number of isolates																												
KP899852	1	T	.	.	C	.	.	.	.	.	.	.	.	.	.	.	.	.	.	T	C	T	C	.	.	.	G	.	.
KP899853	1	.	Y	Y	Y	.	.	.	.	.	Y	.	Y	.	.	.	.	.	.	.	.	.	C	.	.	.	.	.	.
KP899854	1	.	.	Y	.	Y	.	.	Y	.	.	.	.	.	.	.	.	.	.	Y	.	.	C	.	.	.	R	.	.
KP899855	1	.	.	.	Y	Y^1^	R	.	.	.	Y	.	Y	.	.	.	.	.	.	.	Y	.	C	.	.	.	G	.	.
KP899856	1	.	.	.	Y	.	.	.	.	.	Y	.	Y	.	.	.	.	.	.	Y	Y	.	C	Y	.	.	R	.	.
KP899857	1	.	.	.	Y	.	.	.	.	.	.	.	T	.	.	.	.	.	.	Y	.	.	Y	.	.	.	R	.	.
KP899858	1	.	.	.	.	C	A	.	**S**	.	.	.	.	.	.	.	.	.	.	.	.	.	.	.	.	.	.	.	.
KP899859	1	.	.	.	.	C	R	.	.	.	.	.	.	.	.	.	.	.	.	.	.	.	C	.	.	.	R	.	.
KP899860	1	.	.	.	.	C	.	A	.	A	.	.	.	.	.	.	.	.	.	.	.	.	C	T	.	.	.	.	.
KP899861	1	.	.	.	.	C	.	.	Y	.	Y	.	Y	.	.	.	.	Y	.	Y	C	Y	C	.	.	.	R	.	.
KP899862	1	.	.	.	.	Y	.	.	Y	.	Y	.	Y	.	.	.	.	Y	.	.	Y	.	C	.	.	.	.	.	.
KP899863	1	.	.	.	.	Y	.	.	Y	.	Y	.	Y	.	.	.	.	.	.	.	.	.	C	.	.	.	.	.	.
KP899864	1	.	.	.	.	Y	.	.	.	.	.	.	.	.	.	.	.	.	.	.	Y	.	C	.	.	.	R	.	.
KP899865	1	.	.	.	.	C	.	.	.	.	.	.	T	.	.	A^2^	.	.	.	.	.	.	C	.	.	T	.	.	.
KP899866	1	.	.	.	.	Y	.	.	.	.	.	.	.	R	.	.	.	.	.	.	.	.	C	.	.	.	.	.	.
KP899867	1	.	.	.	.	C	.	.	.	.	.	.	.	.	.	.	Y	.	.	Y	Y	.	C	Y	Y	.	.	.	.
KP899868	1	.	.	.	.	Y	.	.	.	.	.	.	.	.	.	.	.	.	.	Y	Y	.	Y	Y	.	.	R	R	Y
KP899869	2	.	.	.	.	C	.	.	.	.	.	.	.	.	.	.	.	.	.	.	.	.	C	.	.	.	.	.	.
KP899870	1	.	.	.	.	C	.	.	.	.	.	.	.	.	.	.	.	.	.	.	.	.	C	Y	Y	.	R	.	.
KP899871	1	.	.	.	.	.	.	.	.	.	T	.	.	.	.	.	.	.	.	.	.	.	C	.	.	.	.	.	.
KP899872	1	.	.	.	.	.	.	.	.	.	.	Y	Y	.	**S**	.	.	.	.	.	.	.	C	.	.	.	R	.	.
KP899873	1	.	.	.	.	.	.	.	.	.	.	.	.	.	.	.	.	.	.	.	C	.	C	.	.	.	G	.	.
KP899874	1	.	.	.	.	.	.	.	.	.	.	.	.	.	.	.	.	.	Y	.	.	.	C	.	.	.	.	.	.

R: A/G; S: G/C; Y: C/T.

^1^ p.M63X.

^2^ p.A99T.

In addition, multiple sequence alignment analyses were also independently performed with the 12 *G*. *duodenalis* isolates harbouring unclear BIII/BIV sub-assemblages and each of the reference sequences AF069059 (BIII) and L40508 (BIV) (S2 and S3 Tables). The obtained results demonstrated the presence of 11 sub-types varying by three to 10 SNPs that were deposited in GenBank under accession numbers KP899875 to KP899885. Depending on the reference sequence considered, mixed bases (double peaks) were responsible for 37.3% to 42.4% of the SNPs detected. Less than 2% of the SNPs identified were due to transversion mutations (S2 and S3 Tables).

**Fig 3:**
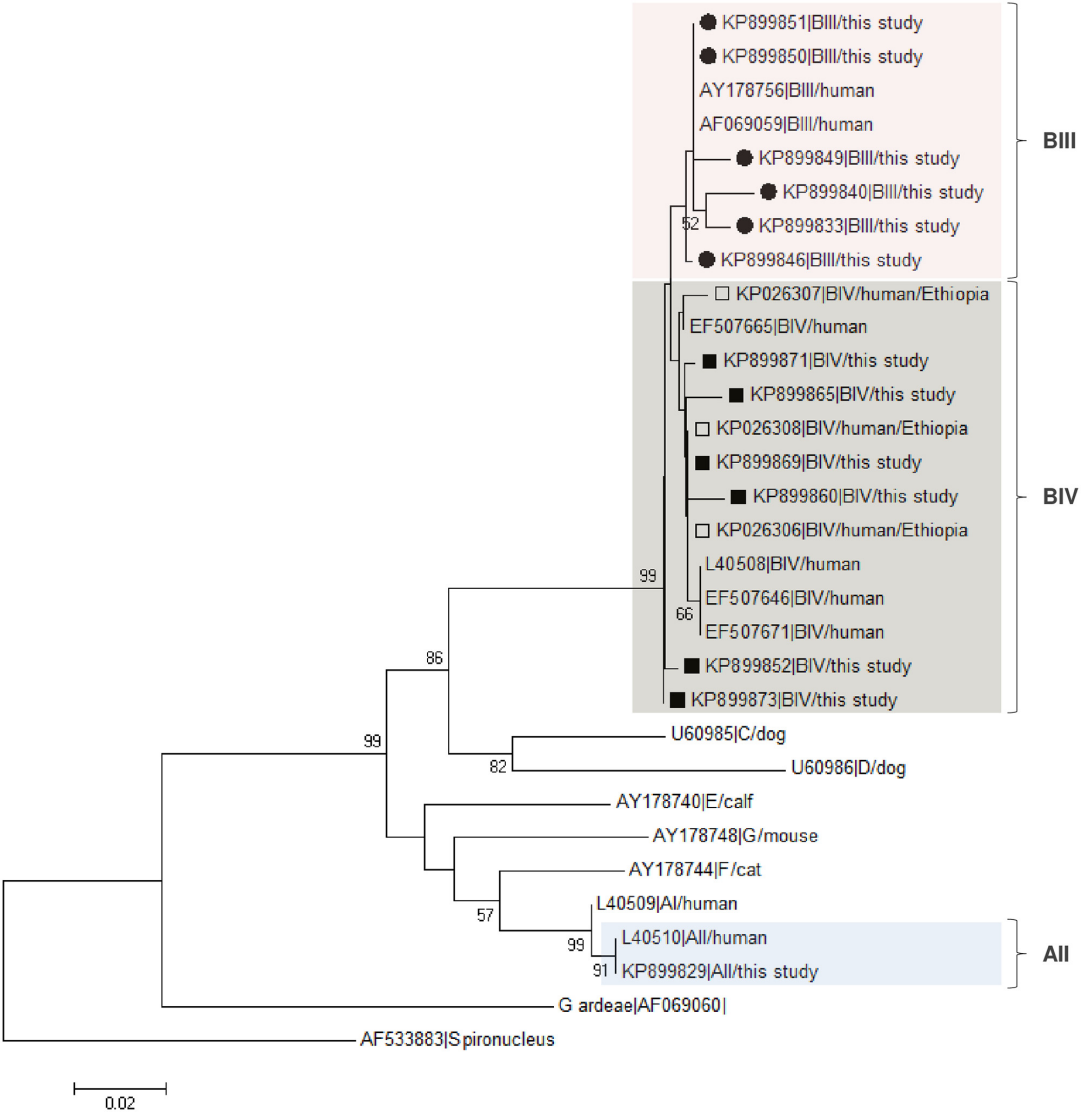
Evolutionary relationships among assemblages of *G*. *duodenalis* at the *GDH* locus inferred by a Neighbor-Joining analysis of the nucleotide sequence covering a 403-bp region (positions 80 to 467 of GenBank accession number L40508) of the gene. The percentage of replicate trees in which the associated taxa clustered together in the bootstrap test (1,000 iterations) is indicated next to the branches. Bootstrap values lower than 50% were not displayed. The evolutionary distances were computed using the Kimura 2-parameter method. The rate variation among sites was modelled with a gamma distribution (shape parameter = 2). Filled circles and squares represent BIII and BIV sequences, respectively, from this study. Open squares indicate BIV sequences previously reported in human isolates from Ethiopia [see ref. 22]. *Spironucleus vortens* was used as outgroup taxa.

Fig 3 shows the phylogenetic tree obtained with the Neighbour-Joining analysis of unambiguous (homozygous) sequences from all the *G*. *duodenalis* sub-assemblages AII, BIII and BIV obtained in this study at the *GDH* marker and the representative reference sequences taken from the NCBI database. No isolates with unclear BIII/BIV genotype results were included in this analysis for clarity purposes. The topology of the produced phylogenetic tree clearly clustered all the BIII and BIV isolates in two discrete groups, respectively.

### Molecular characterization of *G*. *duodenalis* isolates at the *BG* locus

Good quality sequencing data were also available for the 53 *BG*-PCR products obtained. Alignment analyses revealed the presence of two sub-types of *G*. *duodenalis* assemblage A. One sub-type (n = 2) was identified as AII and showed 100% similarity with a stretch of sequence of 487 bp comprising positions 103–589 of reference sequence AY072723. The other sub-type (n = 6) was characterized as AIII and its nucleotide sequence was identical to that comprising positions 103–575 of reference sequence AY072724. Representative sequences of these sub-types were submitted to GenBank under accession numbers KP899830 and KP899831.

Alignment analyses of the 45 *G*. *duodenalis* isolates previously assigned to assemblage B with reference sequence AY072727 resulted in the identification of a 466-bp fragment equivalent to positions 108–573 of AY072727. A total of 25 (7 novel and 18 known) sub-types were differentiated (Table 5). One of them (n = 4) exhibited 100% identity with the reference sequence used, whereas the remaining 24 differed by one to four SNPs with it and were deposited in GenBank under the accession numbers KP899886 to KP899910. All SNPs identified corresponded to transition mutations, with double peaks being responsible for 45.9% (28/61) of them. Only two of the point mutations found were associated with amino-acid changes in the protein sequence (Table 4).

**Table 5:** Diversity and frequency of single-nucleotide polymorphisms displayed by *Giardia duodenalis* assemblage B isolates at the beta giardin locus (partial sequence between positions 108 to 573) identified in the present study. Novel sub-types were shown underlined. Point mutations inducing amino-acid substitutions were marked as superscript indicating the amino-acid change.

		Nucleotide at position of reference sequence AY072727																								
		120	156	165	183	228	240	282	294	309	312	333	339	343	375	378	415	429	432	435	450	474	507	519	540	543
		C	C	C	A	A	T	C	C	C	T	C	C	A	C	C	C	G	A	C	C	C	C	T	C	C
Sub-type	Number of isolates																									
KP899886	1	.	.	.	.	.	.	.	.	.	.	.	.	.	.	.	.	.	.	.	.	.	.	.	.	.
KP899887	1	T	.	.	.	.	.	.	.	.	.	.	.	.	.	.	.	.	.	.	.	.	.	.	.	.
KP899888	2	.	T	.	.	.	.	.	.	.	.	.	.	.	.	.	.	.	.	.	Y	.	.	.	.	.
KP899889	3	.	.	T	.	.	.	.	.	.	.	.	.	.	.	.	.	.	.	.	.	.	.	.	.	.
KP899890	3	.	.	Y	.	.	.	.	.	T	.	.	.	.	.	.	.	.	.	.	.	.	.	.	.	.
KP899891	1	.	.	Y	.	.	.	.	.	Y	.	.	.	.	.	.	.	.	.	.	.	.	.	.	.	T
KP899892	1	.	.	T	.	.	.	.	.	T	.	.	.	.	T	.	.	.	.	.	.	.	.	.	.	T
KP899893	1	.	.	Y	R	.	.	Y	.	.	.	.	.	.	.	.	.	.	.	.	Y	.	.	.	.	.
KP899894	1	.	.	T	R	.	.	.	.	T	.	.	.	.	.	.	.	.	.	.	.	.	.	.	.	Y
KP899895	1	.	.	Y	.	.	Y	.	.	Y	.	.	.	.	.	.	.	.	.	.	.	.	.	.	.	.
KP899896	1	.	.	T	.	.	.	.	.	T	.	.	.	.	Y	.	.	.	.	.	.	.	.	.	.	.
KP899897	1	.	.	Y	.	.	.	.	.	.	Y	.	.	.	.	.	.	.	.	.	.	.	.	.	.	.
KP899898	1	.	.	T	.	.	.	.	.	.	.	T	.	R^1^	.	.	.	.	.	.	.	.	.	.	T	.
KP899899	1	.	.	T	.	.	.	.	.	.	.	.	.	.	.	T	.	A	G	.	.	.	.	.	.	.
KP899900	1	.	.	.	G	.	.	.	.	.	.	.	.	.	.	.	.	.	.	.	.	.	.	.	.	.
KP899901	1	.	.	.	R	.	.	.	.	Y	.	.	.	.	.	.	.	.	.	.	.	Y	.	.	.	.
KP899902	1	.	.	.	.	R	.	.	.	T	.	.	.	.	.	.	.	.	.	Y	.	.	.	.	.	.
KP899903	1	.	.	.	.	.	C	.	.	T	.	.	.	.	.	.	.	.	.	.	.	.	.	C	.	.
KP899904	1	.	.	.	.	.	.	.	Y	Y	.	.	.	.	.	.	.	.	.	.	.	.	.	.	.	.
KP899905	1	.	.	.	.	.	.	.	T	T	.	.	.	.	.	.	.	.	.	.	.	.	T	.	.	.
KP899906	13	.	.	.	.	.	.	.	.	Y	.	.	.	.	.	.	.	.	.	.	.	.	.	.	.	.
KP899907	1	.	.	.	.	.	.	.	.	T	.	Y	.	.	.	.	.	.	.	.	.	.	.	.	.	.
KP899908	1	.	.	.	.	.	.	.	.	T	.	.	T	.	.	.	.	.	.	.	.	.	.	.	.	.
KP899909	1	.	.	.	.	.	.	.	.	Y	.	.	.	.	.	.	Y	.	.	.	Y^2^	.	.	.	.	.
KP899910	1	.	.	.	.	.	.	.	.	.	.	.	.	.	.	.	.	.	.	.	.	.	.	C	.	.

R: A/G; Y: C/T

^1^ p.T115A.

^2^ p.L139X.

**Fig 4:**
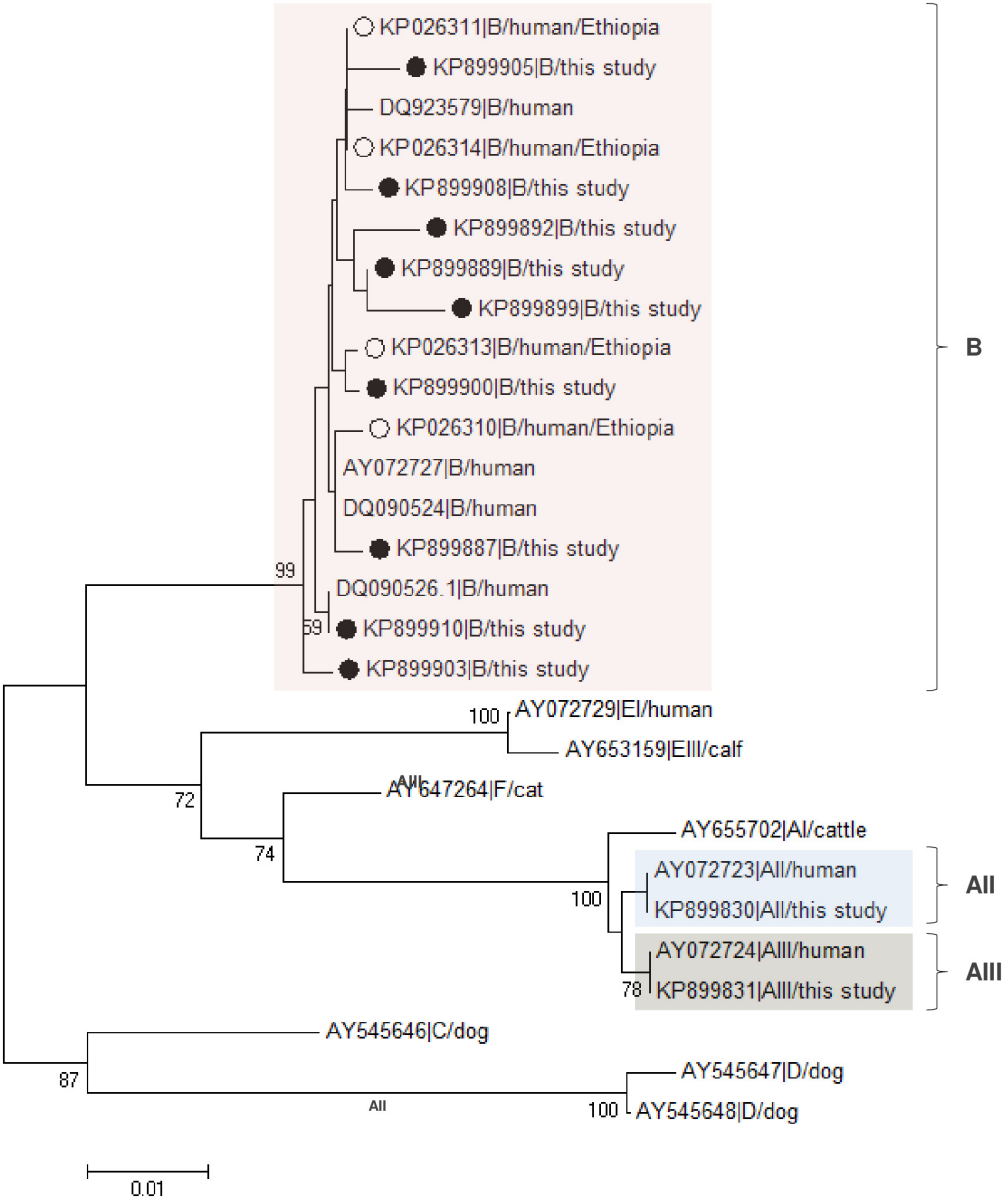
Evolutionary relationships among assemblages of *G*. *duodenalis* at the *BG* locus inferred by a Neighbor-Joining analysis of the nucleotide sequence covering a 487-bp region (positions 108 to 573 of GenBank accession number AY072727) of the gene. The percentage of replicate trees in which the associated taxa clustered together in the bootstrap test (500 iterations) is indicated next to the branches. Bootstrap values lower than 50% were not displayed. The evolutionary distances were computed using the Kimura 2-parameter method. The rate variation among sites was modelled with a gamma distribution (shape parameter = 2). Filled circles represent B sequences from this study, whereas open circles indicate B sequences previously reported in human isolates from Ethiopia (see ref. [22]).No outgroup taxa was used as beta-giardin is a *Giardia*-specific structural protein.

Fig 4 displays the phylogenetic tree constructed by the Neighbor-Joining method to compare unambiguous (homozygous) sequences assigned to assemblage B or sub-assemblages AII and AIII in this study at the *BG* locus with appropriate reference sequences. Our assemblage B isolates formed a well-supported cluster clearly differentiable from other *G*. *duodenalis* assemblages. Similarly, AII and AIII sub-assemblages clustered in independent, but closely related, subsets.

### PCR amplification and molecular characterization of *Cryptosporidium* spp. isolates

The presence of *Cryptosporidium* spp. was detected by PCR in 4.6% (95% CI: 2.5–6.7%) of the stool samples examined. Because of the low number of positive samples found, *Cryptosporidium* infections were not significantly associated to any of the variable considered in this study, including school of origin, gender or group of age of the children examined (data not shown). *Cryptosporidium* species identification was defined on the basis of the subsequent nucleotide sequence analysis of the *SSU* rRNA gene fragment. Out of the 18 *Cryptosporidium* isolates initially amplified at this locus, sequence data of acceptable quality was only produced for five isolates, which were assigned to *C*. *hominis* (n = 2), *C*. *parvum* (n = 1), and *C*. *viatorum* (n = 2). The remaining 13 *Cryptosporidium* isolates were only typed at the genus level. Attempts to classify *C*. *hominis* and *C*. *parvum* samples to the sub-type level based on the specific amplification of the hypervariable *GP60* gene failed repeatedly.

Multiple sequence alignment analysis of one of the obtained *C*. *viatorum* isolates with the reference sequence JX644908 for the partial *SSU* rRNA gene generated a 721-bp fragment corresponding to positions 88–808 of JX644908 and resulted in perfectly matching nucleotide sequences.

## Discussion

Ethiopia harbours most of the NTDs included in the WHO´s list, including ascariasis, trichuriasis, hookworm infections, trachoma, onchocerciasis, and lymphatic filariasis, among others [36,37]. Whereas the disease burden resulting from NTD helminthic infections is reasonably well known, there is a dearth of information regarding protozoan parasitic infections [6,36]. Both giardiasis and cryptosporidiosis, although not formally considered as NTDs, belong to the group of poverty-related infectious diseases that impair the development and socio-economic potential of infected individuals in low income countries including Ethiopia [6]. Human infections by *G*. *duodenalis* and *Cryptosporidium* spp. have been reported in a number of community-based epidemiological surveys in four out of the nine Ethiopian administrative regions, including Amhara [8,20], Harari [26], Oromia [21,22,24,25], the Southern Nations, Nationalities, and People´s Region [10,16,24], and the two chartered cities of Addis Ababa [23] and Dire Dawa [17]. Although geographically restricted, this information strongly suggests that human giardiasis and cryptosporidiosis are endemic in the country. Perhaps more worrying is the general lack of molecular data concerning the *G*. *duodenalis* and *Cryptosporidium* species and sub-types currently circulating in Ethiopian human and animal populations [22,28,29], as this information is essential to ascertain the transmission dynamics of these pathogens and to identify potential animal reservoirs of infection to humans [11,12,38].

A striking finding in this study is the high prevalence of giardiasis detected in the Bahir Dar district, where one in two school children was infected with *G*. *duodenalis*. This infection rate is considerably more elevated than those (11.7–35.3%) described in similar epidemiological surveys conducted in other Ethiopian regions [10,17,18]. This marked variation can be attributed, at least partially, to the different diagnostic sensitivities of the methods (PCR *vs* conventional microscopic examination) used in these studies, although other factors including access to safe drinking water, sanitation and hygiene practices, and contact with production or domestic animals are also likely to make an impact (see below). Importantly, a low proportion (2.8%) of real-time PCR-positive samples were associated to Ct values >36, raising doubts on whether they reflect true or false positive results. In this regard, a previous comparative study conducted in our laboratory evidenced that real-time PCR provides a superior diagnostic sensitivity than direct fluorescence [39]. Concordant results between both methods were observed for 73.9% of the positive samples tested, including some *G*. *duodenalis* isolates with real-time PCR Ct values up to 38. Overall, these data seem to suggest that most of our real-time PCR positive results represent true positive amplifications. Interestingly, females were significantly more at risk of having giardiasis than males. This gender bias in infection has also been documented in other (but not all) prevalence-based epidemiological studies in Ethiopia [10,16,20]. Occupational and behavioural factors such as fetching water from an open water source or indoor activities have been proposed as potential risk factors contributing to this apparent differential exposure to giardiasis, although statistical significance was lacking [16]. Additionally, our analysis of the *G*. *duodenalis* prevalence data stratified by age group or school of origin did not show significant differences, indicating that an elevated transmission intensity of giardiasis with repeated and/or cumulative infections may take part in this geographical area of Ethiopia. Consistent with this epidemiological speculation a positive correlation between real-time PCR Ct values and age was observed in children with giardiasis, with older individuals consistently providing higher Ct values than younger individuals. This finding supports the notion that previous exposure to *G*. *duodenalis* triggers a noticeable adaptive immune response in infected subjects in endemic areas leading to reduced risk of re-infection or reduced development of symptoms in secondary infections [40].

Perhaps the most important contribution of this study is the considerable number of *G*. *duodenalis*-positive samples successfully typed at the *GDH* and/or the *BG* loci, with MLG data available for 48 field isolates. This is in spite of the fact that a comparatively low (24.5–33.8%) percentage of the real-time PCR products that tested positive for *G*. *duodenalis* could be amplified at the *GDH*/*BG* loci. This apparent limitation was a direct consequence of the inherent features of the molecular methods used. Because of the multi-copy nature of the *SSU* rRNA gene, the real-time PCR assay used for the specific detection of *G*. *duodenalis* infections was expected to exhibit a much higher sensitivity than the nested or semi-nested PCR protocols used for genotyping purposes, which relied on the amplification of single-copy genes such as *GDH* and *BG*. Therefore, low intensity infections of *G*. *duodenalis* would be only detectable by real-time PCR (with associated high Ct values) but neither by *GDH*-PCR nor *BG*-PCR. In addition, higher amplification rates at the *GDH* marker compared to the *BG* marker were consistently produced. This difference may be influenced by the initial design of the primer sequences used in these PCR protocols. Thus, whereas the *GDH*-PCR was based on degenerated primers covering most possible nucleotide variations among assemblages A–F [32], the *BG*-PCR used set of primers specifically chosen to match conserved regions of assemblages A, B, and E [33]. Indeed, multiple alignment analyses of representative *GDH* and *BG* sequences with the respective primer sequences revealed important intra- and inter-assemblage variations at the nucleotide level in the compared stretch (S4 Table). For instance, primers G7_F and G759_R used to amplify *BG* sequences may fail to detect a number of B isolates because of the presence of three primer-template mismatches (S4 Table). Another potential limitation of this study is that both the *GDH* and *BG* genes are located on chromosome 4 of *G*. *duodenalis*, a fact that may influence the robustness of our sub-typing results. It could be argued that, ideally, assigned sub-assemblages should be confirmed at different loci located on different chromosomes.

Taking advantage of the large panel of *G*. *duodenalis* samples molecularly characterized at the *GDH* and *BG* genes, we conducted a detailed analysis of the genetic diversity and variability at the nucleotide level of these isolates. Our data clearly show that assemblage B was the predominant genotype circulating in the children population under study, accounting for 82% of all the isolates typed, with the remaining 18% being assigned to assemblage A. These results, together with the absence of the animal-specific assemblages C-H, were in line with those typically reported in developing countries [11,12], including Ethiopia [22,28]. The MLG scheme adopted here proved its usefulness for confirming assigned *G*. *duodenalis* sub-types, particularly within assemblage B. Both assemblages A and B are widely accepted to have a broad range of suitable hosts with the potential to be transmitted zoonotically [41]. In this regard, earlier epidemiological studies in Ethiopia have proposed a role for cattle [18] and pet animals [28] as sources of human giardiasis, although the lack of genotyping data from domestic animals species in these surveys did not allow confirming the extent of this hypothesis.

An extremely high degree level of genotypic variation in the form of mixed base polymorphisms were observed within assemblage B (but not within assemblage A) at both the *GDH* and *BG* loci, as previously documented in a number of molecular epidemiological surveys worldwide [42–44]. Di-nucleotide (heterozygous) sites accounted for a large proportion (45.9% to 67.9%) of the SNPs detected and contributed to an abundance of sequence types harbouring private di-nucleotide sites only. Taken together, these findings may be suggestive of poor sequencing reactions. This does not seem to be the case in our study, where all electropherograms were visually inspected and verified for sequencing quality and signal strength. Indeed, most of the heterozygous sites (double peaks) detected were observed in both forward and reverse sequences, although not always with equal intensities. Consequently, the vast majority of the isolates characterized corresponded to different genotypic variants of *G*. *duodenalis* sub-assemblages BIII and BIV. It is worth noticing that the opposite phenomenon has been documented by our laboratory in a recent survey based on the same molecular tools and targeting symptomatic individuals with giardiasis in Spain [39], a developed country with lower prevalence rates of the disease [45]. In that study we demonstrated that most of the BIII-BIV isolates identified belonged to a restricted number of *G*. *duodenalis* genotypes. Additionally, the proportion of conflicting typing results AII/AIII and BIII/BIV observed in these Ethiopian and Spanish populations were 23.1% and 10.5%, respectively. Taken together these data strongly suggest that the genetic diversity of *G*. *duodenalis* in a given area or human population is highly dependent on the infection pressure and the transmission intensity of the parasite.

As a result, a large amount of B-type nucleotide sequences representing novel and existing variants of sub-assemblages were identified. The vast majority (>98%) of the nucleotide substitutions detected (including heterogeneous positions) corresponded to transitional mutations, less likely to result in amino-acid substitutions and, therefore, more likely to persist as silent substitutions [46–48]. This is also the case of our molecular data, where very few (7/259) SNPs altered the deduced amino-acid sequences of the *GDH* and *BG* genes.

Two potential mechanisms have been proposed to explain the large molecular variability observed, namely the presence of true mixed infections with different *G*. *duodenalis* genotypes and the occurrence of genetic recombination leading to allelic sequence heterozygosity (ASH), that is, the sequence dissimilarity between different alleles of the same gene [12,49–51]. Unfortunately, the relative contribution of each of these mechanisms to the sequence heterogeneity found remains largely unknown due to the lack of conclusive experimental evidence. The increasing use of MLG approaches based on assemblage-specific PCR assays has revealed that mixed infections are more common than initially thought, particularly in endemic areas of low-income countries [11,12]. Interestingly, no mixed infections involving different *G*. *duodenalis* assemblages were detected in the present study. This finding seems to support the role of recombination as the main source of the genetic variation observed at the sub-assemblage level a fact that should be confirmed in future epidemiological studies with additional molecular markers.

Regarding the second possibility, *Giardia* is presumed to be a strictly clonal (asexual) organism based on the lack of an observed sexual cycle or gametes [49]. Under this assumption, the nuclei of the parasite are expected to have independently accumulated mutations leading to divergence of haplotypes over time, and therefore, relatively elevated levels of ASH [11,51]. However, the unexpected finding that *G*. *duodenalis* assemblage A has a very low (<0.01%) level of ASH [49] strongly suggested the presence of some sort of genome-homogenising mechanism. Diplomixis (fusion of the two nuclei of *G*. *duodenalis* in the cyst stage) leading to gene conversion or crossing-over events can most likely achieve this effect [52], raising the question of whether *Giardia* is also capable of sexual reproduction. Indeed, evidence of genetic recombination has been demonstrated at the cellular level in cultured assemblage B trophozoites and in cysts from clinical samples [53], and at the population level within sub-assemblages BIII and BIV [47]. However, somewhat conflicting results are currently available regarding the potential exchange of genetic material between genotypes of different *G*. *duodenalis* assemblages [54,55], an issue that clearly needs further investigation. In practical terms, it has been recently proposed that ASH events at the cellular level in concurrence with mixed infections involving different assemblage B sub-genotypes may represent a convincing explanation for the elevated sequence heterogeneity commonly reported on *G*. *duodenalis* assemblage B isolates in field surveys [53]. The molecular data presented here may provide indirect evidence support this epidemiological scenario.

Cryptosporidiosis cases were only identified in 4.6% of the school children population under study, an infection rate somewhat lower than those (5.2–12.2%) previously reported in other Ethiopian paediatric populations [16–18]. This discrepancy is further exacerbated when considering that our prevalence data were obtained by PCR, a methodology considerably more sensitive than the conventional microscopy with Ziehl–Neelsen staining used in other epidemiological studies. This fact seems to suggest that the ecological, environmental, and demographic conditions of the Bahir Dar district were not particularly suited for the transmission of *Cryptosporidium* infections at the community level. Another interesting finding was the identification of *Cryptosporidium viatorum* in two of our field isolates. *C*. *viatorum* is a novel *Cryptosporidium* species initially described in 2012 among symptomatic travellers (*n* = 10) returning to UK from the Indian sub-continent [56]. Since then, human infections caused by *C*. *viatorum* have been only documented in two Swedish patients [57,58], and ten HIV patients in Ethiopia [59] and an additional one in Nigeria [60].

## Conclusions

This community-based molecular epidemiological study demonstrated that *G*. *duodenalis* and *Cryptosporidium* spp. infections were common in elementary and middle school aged children in the Bahir Dar district of Ethiopia. The particularly high prevalence rate of *G*. *duodenalis* found was indicative of an elevated transmission intensity of the parasite translating into repeated and cumulative infections. Consistent with this epidemiological scenario was the identification of an elevated degree of genetic diversity at the nucleotide level within assemblage B. We believe that the later finding provides relevant molecular epidemiological evidence supporting the hypothesis of the occurrence of intra-assemblage recombination in *G*. *duodenalis*. Finally, because human infections by *G*. *duodenalis* and *Cryptosporidium* spp. are largely dependent on the socioeconomic and educational status of a given community (a feature shared with many other infectious diseases including NTDs) there is an urgent need to design and implement integrative control programs aiming to improve sanitation and health conditions in endemic areas of developing countries in order to optimize intervention strategies and available resources.

## Supporting Information

S1 Table List of oligonucleotides used for the molecular identification and characterization of *Giardia duodenalis* and *Cryptosporidium* spp. in this study. (DOCX)

S2 Table Diversity and frequency of single-nucleotide polymorphisms displayed by mixed infections of *Giardia duodenalis* sub-assemblages BIII+BIV isolates at the glutamate dehydrogenase locus identified in the present study. (DOCX)

S3 Table Diversity and frequency of single-nucleotide polymorphisms displayed by mixed infections of *Giardia duodenalis* sub-assemblages BIII+BIV isolates at the glutamate dehydrogenase locus identified in the present study. (DOCX)

S4 Table Multiple alignment of individual sense and antisense (5'-3') primer sequences used to amplify partial fragments of the glutamate dehydrogenase (*GDH*) and ß-giardin (*BG*) genes of *Giardia duodenalis* with representative sequences of assemblages A and B retrieved from GenBank showing the number of mismatches allowed in each primer sequence. Reference sequences used in this study (highlighted in bold) were also included in the analyses.
(XLS)
