# Supplementary material for: Prevalence and Genetic Diversity of Giardia duodenalis and Cryptosporidium spp. among School Children in a Rural Area of the Amhara Region, North-West Ethiopia
Source: PLoS One. 2016 Jul 28;11(7):e0159992. doi: 10.1371/journal.pone.0159992 (PMC4965151; doi:10.1371/journal.pone.0159992)
Supplement: S1 Table — (DOCX) [file pone.0159992.s001.docx]

**S1 Table.**

List of oligonucleotides used for the molecular identification and characterization of *Giardia duodenalis* and *Cryptosporidium* spp. in this study.

| **Target organism** | **Locus** | **Oligonucleotide** | **Sequence (5´–3´) and labels** | **Reference** |
| --- | --- | --- | --- | --- |
| *Giardia duodenalis* | *SSU* rRNA | Primer Gd-80F | GACGGCTCAGGACAACGGTT | 31 |
| *Giardia duodenalis* | *SSU* rRNA | Primer Gd-127R | TTGCCAGCGGTGTCCG | 31 |
| *Giardia duodenalis* | *GDH* | Primer GDHeF | TCAACGTYAAYCGYGGYTTCCGT | 32 |
| *Giardia duodenalis* | *GDH* | Primer GDHiF | CAGTACACCTCYGCTCTCGG | 32 |
| *Giardia duodenalis* | *GDH* | Primer GDHiR | GTTRTCCTTGCACATCTCC | 32 |
| *Giardia duodenalis* | *BG* | Primer G7-F | AAGCCCGACGACCTCACCCGCAGTGC | 33 |
| *Giardia duodenalis* | *BG* | Primer G759-R | GAGGCCGCCCTGGATCTTCGAGACGAC | 33 |
| *Giardia duodenalis* | *BG* | Primer G99-F | GAACGAACGAGATCGAGGTCCG | 33 |
| *Giardia duodenalis* | *BG* | Primer G609-R | CTCGACGAGCTTCGTGTT | 33 |
| *Cryptosporidium* spp. | *SSU* rRNA | Primer 18SiCF2 | GACATATCATTCAAGTTTCTGACC | 34 |
| *Cryptosporidium* spp. | *SSU* rRNA | Primer 18SiCR2 | CTGAAGGAGTAAGGAACAACC | 34 |
| *Cryptosporidium* spp. | *SSU* rRNA | Primer 18SiCF1 | CCTATCAGCTTTAGACGGTAGG | 34 |
| *Cryptosporidium* spp. | *SSU* rRNA | Primer 18SiCR1 | TCTAAGAATTTCACCTCTGACTG | 34 |
