# Supplementary material for: Prevalence and Genetic Diversity of Giardia duodenalis and Cryptosporidium spp. among School Children in a Rural Area of the Amhara Region, North-West Ethiopia
Source: PLoS One. 2016 Jul 28;11(7):e0159992. doi: 10.1371/journal.pone.0159992 (PMC4965151; doi:10.1371/journal.pone.0159992)
Supplement: S2 Table — (DOCX) [file pone.0159992.s002.docx]

**S2 Table.**

**Diversity and frequency of single-nucleotide polymorphisms displayed by conflicting genotype results of sub-assemblages BIII/BIV of *Giardia duodenalis* at the glutamate dehydrogenase locus (partial sequence between positions 42 to 444) identified in the present study.** Sequence AF069059 (BIII) has been used as reference. Transversion mutations were highlighted in bold.

|  |  | **Nucleotide at position of reference sequence AF069059 (BIII)** | | | | | | | | | | | | | | | | | | | | |
| --- | --- | --- | --- | --- | --- | --- | --- | --- | --- | --- | --- | --- | --- | --- | --- | --- | --- | --- | --- | --- | --- | --- |
|  |  | **99** | **147** | **149** | **150** | **189** | **219** | **225** | **237** | **288** | **294** | **309** | **316** | **330** | **336** | **351** | **372** | **375** | **387** | **396** | **402** | **426** |
|  |  | **C** | **T** | **C** | **G** | **G** | **T** | **G** | **T** | **T** | **C** | **C** | **C** | **C** | **C** | **C** | **G** | **C** | **C** | **C** | **G** | **T** |
| **Sub-type** | **Number of isolates** |  |  |  |  |  |  |  |  |  |  |  |  |  |  |  |  |  |  |  |  |  |
| KP899877 | 1 | Y | Y | . | . | . | Y | . | Y | . | . | Y | . | Y | . | . | . | . | . | Y | R | . |
| KP899881 | 1 | T | Y | . | . | . | Y | . | Y | . | . | . | . | Y | . | . | . | . | . | . | R | . |
| KP899883 | 1 | T | C | Y | . | . | Y | . | . | . | . | Y | . | Y | Y | . | . | . | . | Y | R | Y |
| KP899882 | 1 | T | C | . | A | . | C | . | C | . | . | . | . | . | . | . | A | T | . | . | . | . |
| KP899879 | 2 | T | . | . | . | . | C | . | C | . | . | . | . | . | . | . | . | . | . | . | . | . |
| KP899876 | 1 | T | . | . | . | . | . | A | . | A | . | . | . | T | . | T | . | . | . | . | . | . |
| KP899875 | 1 | T | . | . | . | . | . | . | . | . | Y | . | **M** | Y | . | . | . | . | . | . | R | . |
| KP899884 | 1 | . | C | . | A | . | C | . | C | . | . | . | . | . | . | . | . | . | . | . | . | . |
| KP899885 | 1 | . | . | . | . | A | C | . | C | . | . | . | . | . | . | . | . | . | . | . | A | . |
| KP899878 | 1 | . | . | . | . | . | . | . | . | . | . | T | . | T | . | . | . | . | T | . | A | . |
| KP899880 | 1 | . | . | . | . | . | . | . | . | . | . | . | . | T | . | T | . | . | . | . | A | . |

M: A/C; R: A/G; Y: C/T.
