# Supplementary material for: Prevalence and Genetic Diversity of Giardia duodenalis and Cryptosporidium spp. among School Children in a Rural Area of the Amhara Region, North-West Ethiopia
Source: PLoS One. 2016 Jul 28;11(7):e0159992. doi: 10.1371/journal.pone.0159992 (PMC4965151; doi:10.1371/journal.pone.0159992)
Supplement: S3 Table — (DOCX) [file pone.0159992.s003.docx]

**S3 Table.**

**Diversity and frequency of single-nucleotide polymorphisms displayed conflicting genotype results of sub-assemblages BIII/BIV of *Giardia duodenalis* at the glutamate dehydrogenase locus (partial sequence between positions 78 to 480) identified in the present study.** Sequence L40508 (BIV) has been used as reference. Transversion mutations were highlighted in bold.

|  |  | **Nucleotide at position of reference sequence L40508 (BIV)** | | | | | | | | | | | | | | | | | | | | |
| --- | --- | --- | --- | --- | --- | --- | --- | --- | --- | --- | --- | --- | --- | --- | --- | --- | --- | --- | --- | --- | --- | --- |
|  |  | **135** | **183** | **185** | **186** | **225** | **255** | **261** | **273** | **324** | **330** | **345** | **352** | **366** | **372** | **387** | **408** | **411** | **423** | **432** | **438** | **462** |
|  |  | **T** | **T** | **C** | **G** | **G** | **C** | **G** | **C** | **T** | **C** | **C** | **C** | **T** | **C** | **T** | **G** | **C** | **C** | **C** | **A** | **T** |
| **Sub-type** | **Number of isolates** |  |  |  |  |  |  |  |  |  |  |  |  |  |  |  |  |  |  |  |  |  |
| KP899884 | 1 | C | C | . | A | . | . | . | . | . | . | . | . | C | . | C | . | . | . | . | G | . |
| KP899877 | 1 | Y | Y | . | . | . | Y | . | Y | . | . | Y | . | Y | . | C | . | . | . | Y | R | . |
| KP899885 | 1 | C | . | . | . | A | . | . | . | . | . | . | . | C | . | C | . | . | . | . | . | . |
| KP899880 | 1 | C | . | . | . | . | T | . | T | . | . | . | . | . | . | . | . | . | . | . | . | . |
| KP899878 | 1 | C | . | . | . | . | T | . | T | . | . | T | . | . | . | C | . | . | T | . | . | . |
| KP899882 | 1 | . | C | . | A | . | . | . | . | . | . | . | . | C | . | C | A | T | . | . | G | . |
| KP899883 | 1 | . | C | Y | . | . | Y | . | T | . | . | Y | . | Y | Y | C | . | . | . | Y | R | Y |
| KP899881 | 1 | . | Y | . | . | . | Y | . | Y | . | . | . | . | Y | . | C | . | . | . | . | R | . |
| KP899876 | 1 | . | . | . | . | . | T | A | T | A | . | . | . | . | . | . | . | . | . | . | G | . |
| KP899875 | 1 | . | . | . | . | . | T | . | T | . | Y | . | **M** | Y | . | C | . | . | . | . | R | . |
| KP899879 | 2 | . | . | . | . | . | . | . | . | . | . | . | . | C | . | C | . | . | . | . | G | . |

M: A/C; R: A/G; Y: C/T.
